# Development of ICD 11: changes and challenges

**DOI:** 10.1186/1472-6963-12-S1-I8

**Published:** 2012-11-21

**Authors:** A Zafar, Sharifa Ezat WP

**Affiliations:** 1 International Training Center for Casemix and Clinical Coding, UKM Medical Centre, Cheras Kuala Lumpur, Malaysia; 2 Department of Community Health, UKM Medical Centre, Cheras Kuala Lumpur, Malaysia

## 

Hospitals are data hungry organization, and store tons of patient level data in the patient’s clinical notes. This data cannot be used in the day to day decision making because it is not standardized. That was the rationale that started the movement to standardize the clinical documentation which resulted in the development of clinical coding standards based on disease classification. Disease classification has its roots in the classes of causes of death, which started as early as mid 18^th^ century. The classes for the causes of death was formalized by the International Statistical Congress that later evolved into the International Statistical Institute. International Statistical Institute later collaborated with Health Organization of the League of Nations, the precursor of the World Health Organization to incorporate the classification of morbidities, and later on the role for updating and maintenance was wholly taken over by the WHO.

The last revision (10^th^ Revision) of the International Classification of Disease was carried out in 1989 and is famously known as ICD 10. ICD 10 has served its purpose very well, but with the rapid change in the technologies and way the data is managed in hospitals, it is fast becoming obsolete. Therefore WHO has decided to revise the current version of the ICD 10, and come up with ICD 11 that fulfills all the requirements a modern healthcare system. ICD 11 will be a comprehensive classification that can be used by all the different stakeholders in the healthcare environment, can be integrated into the digital hospital information system and other terminologies such as SNOMED CT. For that purpose WHO has come up with a comprehensive program to involve the different stakeholders in the development of the ICD 11 that hopefully, will be commissioned in 2015.

